# Merkel Cell Polyomavirus Small T Antigen mRNA Level Is Increased following *In Vivo* UV-Radiation

**DOI:** 10.1371/journal.pone.0011423

**Published:** 2010-07-02

**Authors:** Ariane Mogha, Alain Fautrel, Nicolas Mouchet, Na Guo, Sébastien Corre, Henri Adamski, Eric Watier, Laurent Misery, Marie-Dominique Galibert

**Affiliations:** 1 Faculté de Médecine, CNRS UMR 6061 Institut de Génétique et Développement de Rennes, Equipe RTO, Rennes, France; 2 Université de Rennes 1, IFR140 GFAS, Rennes, France; 3 Faculté de Médecine, Université de Rennes 1, Plateforme Histopathologie – BioGenouest IFR140 GFAS, Rennes, France; 4 PROCLAIM, Saint Grégoire, France; 5 CHU Pontchaillou, Service de Dermatologie, Rennes, France; 6 Hopital Sud, Service de Chirurgie Plastique, Rennes, France; 7 EA 4326 Laboratoire de Neurobiologie Cutanée, Université de Brest, Brest, France; 8 Laboratoire de Génomique Médicale, CHU Pontchaillou, Plateforme Transcriptomique GenOuest, Rennes, France; University of Kansas Medical Center, United States of America

## Abstract

Merkel cell carcinoma (MCC) is a rare but aggressive skin cancer involving Merkel cells. Recently, a new human polyomavirus was implicated in MCC, being present in 80% of the samples analyzed. In virus-positive MCC, the Merkel cell polyomavirus (MCPyV) is clonally integrated into the patients DNA, and carries mutations in its large T antigen, leading to a truncated protein. In non-symptomatic tissue MCPyV can reside at very low levels. MCC is also associated with older age, immunosuppression and sun exposure. However, the link with solar exposure remains unknown, as the precise mechanism and steps involved between time of infection by MCPyV and the development of MCC. We thus investigated the potential impact of solar simulated radiation (SSR) on MCPyV transcriptional activity. We screened skin samples of 20 healthy patients enrolled in a photodermatological protocol based on *in vivo*-administered 2 and 4 J/cm^2^ SSR. Two patients were infected with two new variants of MCPyV, present in their episomal form and RT-QPCR analyses on SSR-irradiated skin samples showed a specific and unique dose-dependent increase of MCPyV small t antigen transcript. A luciferase based *in vitro* assay confirmed that small t promoter is indeed UV-inducible. These findings demonstrate that solar radiation has an impact on MCPyV mRNA levels that may explain the association between MCC and solar exposure.

## Introduction

Merkel cell carcinoma (MCC) is a highly aggressive cancer of the skin involving neuroendocrine mechanoreceptor Merkel cells [1], first described by Toker in 1972 [2]. Its incidence has increased sharply over the past twenty years, from 500 to 1500 new cases in 2008 in the United States [3]. With a two-year mortality rate of 28%, MCC is more severe than melanoma. Although it is extremely rare in people younger than 50 years old (<5%), MCC risk increases with age and is eleven times higher for HIV-positive patients [4]. A higher risk is also associated with immuno-deficiency. MCC, like melanoma, is associated with sun exposure and affects preferentially individuals with light-colored skin [5]. Additionally, 36% of MCC involve the face, the most sun-exposed anatomical site.

In 2008, a new polyomavirus called Merkel cell polyomavirus (MCPyV) was identified in 8 of the 10 MCC tumors studied [6]. MCPyV DNA is clonally integrated into the tumor genome and is mutated in the large T (LT) antigen-encoding sequence. Viral integration is proposed to occur early in MCC carcinogenesis, before tumor expansion. Recent studies confirmed the presence of high levels of MCPyV DNA in MCC tumors [7], [8], [9], [10]. These findings are consistent with reports of serology titers [11], [12], [13], whereas low levels have been found in other tissues of healthy controls [6], [8], [14], [15]. Although recent data have significantly advanced our understanding of the pathogenesis of this disease, the molecular events involved in the progression from non-pathogenic viral infection to MCC development remain unclear. Similarly, the precise role of solar radiation, if any, in the context of viral infection, remains to be elucidated.

## Results

### Photodermatological protocol and MCPyV positive-samples

**Figure 1:**
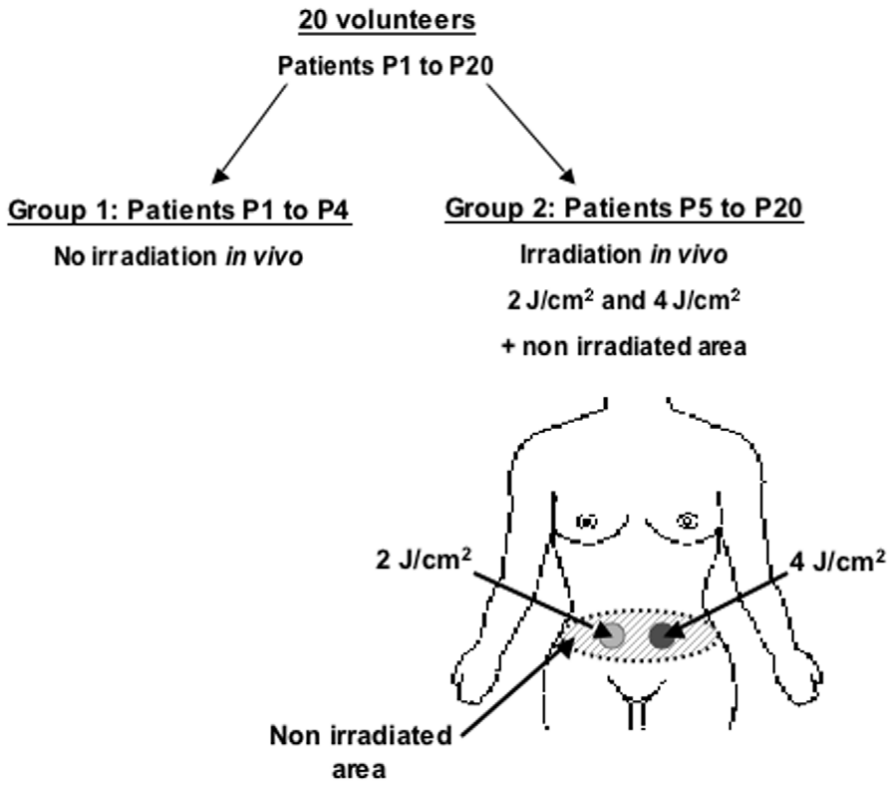
Photobiological protocol. The 20 volunteers were divided into two subgroups. Group 1: patients P1 to P4 did not received in vivo irradiation. Group 2: patients P5 to P20 received 2 J/cm2 and 4 J/cm2 SSR irradiation five hours before abdominal plastic surgery. The irradiated regions were distant one from each other and emitted radiations did not affect the surrounding skin. Around five skin patches were taken from each region.

We established a photodermatological protocol to examine the impact of solar radiation on MCPyV gene transcription in a non-pathogenic context. We thus recruited twenty female volunteers aged between 38 and 60, with phototype II or III skin type, who had been referred for abdominal plastic surgery (Hôpital Sud, Rennes, France). The photodermatological protocol consisted in irradiating the abdominal zone with 2 J/cm^2^ and 4 J/cm^2^ solar-simulated radiation (SRR), five hours before surgery (Fig.1) [16]. The SSR value of 2 J/cm^2^ corresponds to the minimal erythemal dose (MED) for phototype II- skins [17]. Of the twenty patients (P1 to P20) included, four did not undergo irradiation (P1 to P4), constituting a control subgroup. After surgery, skin samples were immediately collected and processed to obtain nucleic acid samples.

**Figure 2:**
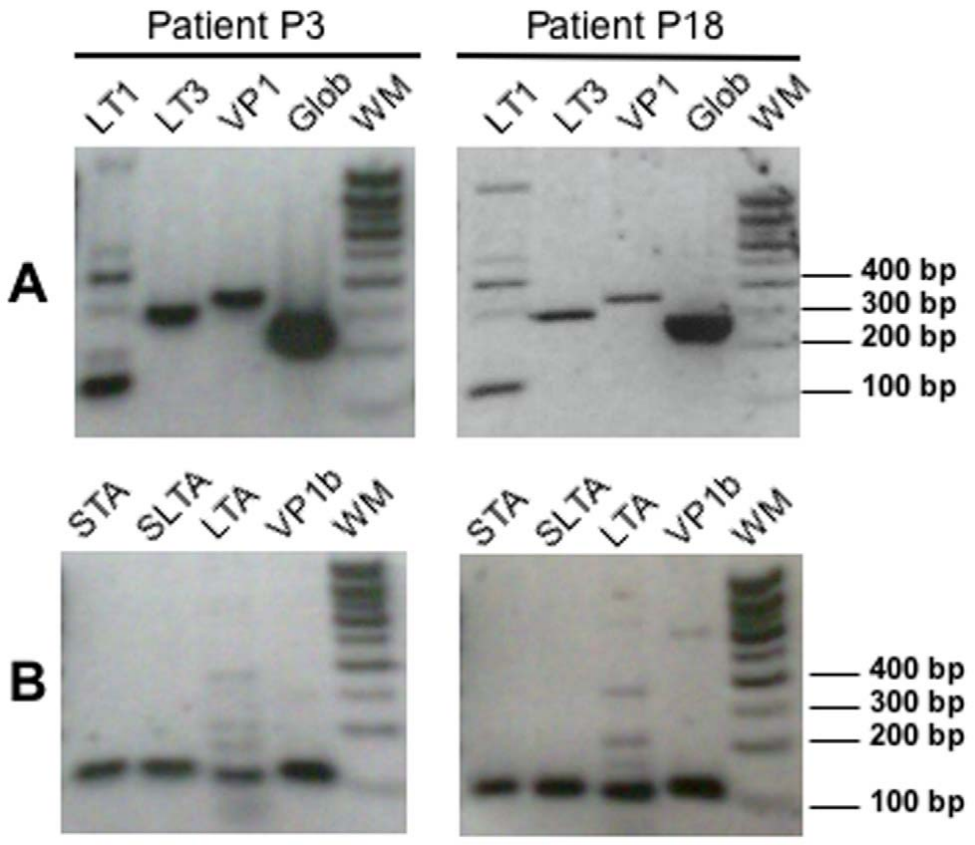
MCPyV positive-samples identified by PCR-screening. Agarose gels showing PCR products obtained from patient P3 and P18 skin sample DNA. **A**, with published (Feng et al., 2008) primers LT1 (expected product at 440 bp), LT3 (308 bp), VP1 (351 bp) and β ~globin (268 bp); WM, weight marker. **B**, with newly designed primers STA (115 bp), SLTA (108 bp), LTA (98 bp) and VP1b (109 bp); WM, weight marker.

We firstly used PCR to screen DNA samples for the presence of MCPyV genome. We used primers specifically targeting the LT antigen (LT1 and LT3) and the capsid structural-protein (VP1), as previously described [6]. LT3 and VP1 primers amplified a specific product of the expected size in two patient samples (P3 and P18), whereas no such specific product was detected with LT1 primers (Figure 2A). We then confirmed the presence of MCPyV in patients P3 and P18 by specific PCR amplification, with newly designed primer-sets targeting distinct regions, of the following MCPyV DNA sequence: the ST antigen (STA), the LT antigen (LTA), a region common to both the ST and LT antigen-sequences (SLTA) and the capsid-sequence (VP1b) (Figure 2B). No viral-specific amplification was observed for the other samples (data not shown). The human β-globin gene, used as a control, was positive for all samples (P1 to P20). Our findings show that patients P3 and P18, of 49 and 42 years old respectively, were infected by MCPyV. This corresponds to 10% of the samples screened, consistent with previous observations and providing further insight to sporadic presence of MCPyV in skin of healthy individuals [14], [18].

### MCPyV genome is episomal in non-pathogenic infected tissue

**Figure 3:**
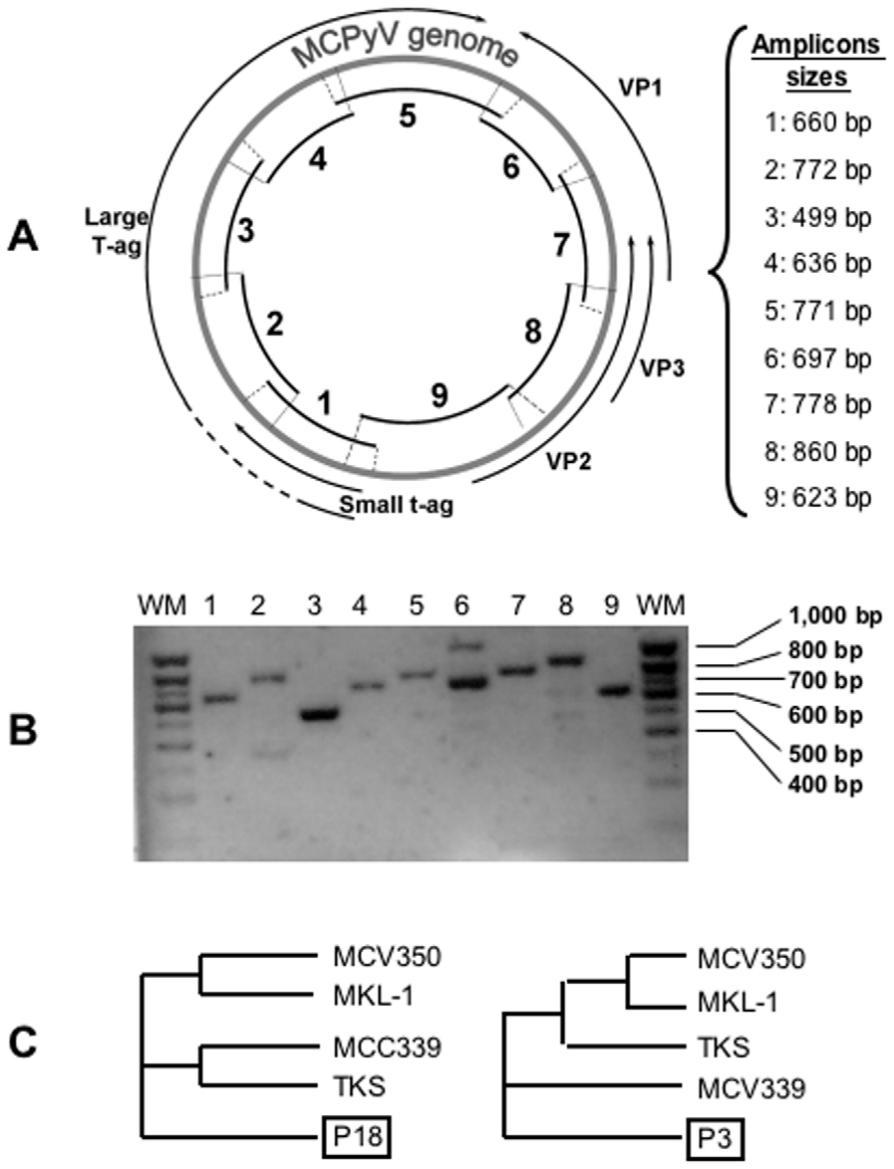
MCPyV genome is episomal in non-pathogenic skin samples. **A**, Schematic representation of the sequenced regions (overlapping sequences 1 to 9) mapped on MCPyV circular DNA. **B**, Agarose gel of the PCR products obtained from patient P18 skin sample DNA using the sequencing primer sets targeting sequence 1 to 9; WM, weight marker. Comparable results are obtained for patient P3 samples. **C**, Cladograms classifying the MCPyV variants found in patients P3 and P18, and variants MCV350, MCV339, MKL-1 and TKS (respective GenBank codes: EU375803, EU375804, FJ173815 and FJ46433.

The status of the MCPyV genome in non-pathogenic samples remains unknown. However, it may be predictive of the clinical outcome or may modulate the potential effect of solar radiation. We thus established a sequencing strategy, to identify potential episomal form and relevant modifications to be compared to the MCPyV sequence that is mutated and clonally integrated into the tumors genome, although some copy virus are suspected to remain episomal [19]. We thus selected conserved regions by MCPyV sequence alignment using publicly available data (GenBank codes: EU375803, EU375804, FJ173815, FJ464337). We then designed overlapping sets of primers spanning the whole genome of the virus (Figure 3A). Using this strategy, we amplified and sequenced the entire viral genome present in both MCPyV-positive patient samples (Figure 3B), suggesting that the virus is predominantly episomal. LT antigen sequences did not carry any mutations that would generate a truncated protein, consistent with its non-integrated state. In addition, we identified several SNPs that were systematically present and specific to each viral genome sequence (ie: P3 or P18). Identical sequence was obtained for every skin samples of each patient. These SNPs distinguish the two newly identified related sequences and demonstrate a level of genetic variability of the virus of about 2% (Figure 3C). These SNPs also explain the absence of specific PCR amplification observed with the LT1 primer set, and may thus also underlie discrepancies observed in the number of MCPyV-negative MCC [9], [20].

We estimated the number of MCPyV copies per cell and their distribution over a 10 cm^2^ skin-area (represented by 11 non-irradiated samples). We thus evaluated MCPyV DNA levels using QPCR with three primer-sets (STA, SLTA and VP1b) and with the human *GAPDH* gene as a reference. We found about 2×10^−4^ to 4×10^−3^ virus DNA copies per *GAPDH* copy for P18 (Table 1), comparable results were obtained with UV-irradiated samples (data not shown). Thus, as in non-MCC skin cancers [7], [8], [21], but unlike MCPyV-positive MCC tumors, non-pathogenic skin infection is associated with very low levels of virus copies, evenly distributed over the abdominal zone, possibly affecting a sub-population of cells. These findings also suggest that MCPyV may be present in non-pathogenic skin areas of patients with MCPyV-positive MCC. This raises the question of what triggers the transforming events. One possibility is that UV-rays play a part in this process.

**Table 1:** MCPyV DNA copy number.

P18 skin samples	MCPyV DNA copies (x10^-4^) for one copy of *GAPDH* gene
Sample 1	8.6
Sample 2	19.0
Sample 3	8.2
Sample 4	11.0
Sample 5	5.1
Sample 6	2.1
Sample 7	9.0
Sample 8	42.4
Sample 9	14.0
Sample 10	5.5
Sample 11	27.2
**Mean**	**13.8**

Relative quantification by QPCR of MCPyV DNA copies in eleven non irradiated skin samples from patient P18; normalization with *GAPDH* gene. Comparable results have been obtained with irradiated skin samples (data not shown).

### The small t antigen mRNA level is modulated following solar exposure

**Figure 4:**
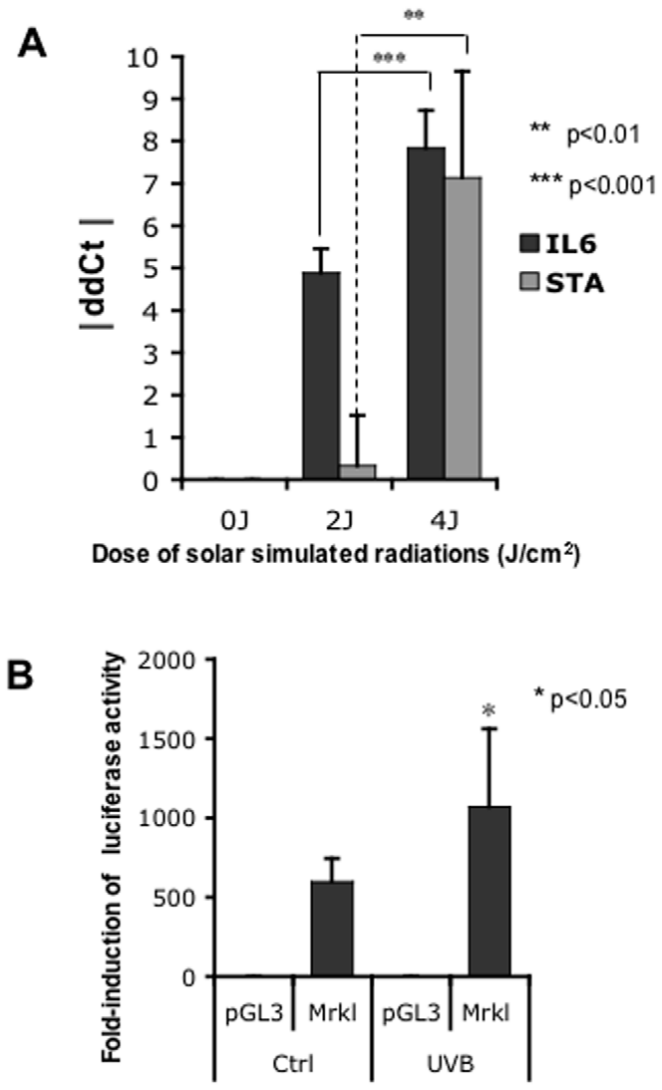
ST antigen mRNA level increases significantly following UV radiation. **A**, Relative quantification of small t antigen (STA) and interleukin 6 (IL6) transcripts by RT-QPCR after a pre-amplification step, using total RNA from patient P18 skin samples, after 2J and 4 J/cm^2^ SSR *in vivo* irradiation, and in control samples. Each histogram bar represents the mean value obtained from six independent QPCR reactions. The Y-axis represents the absolute value of the “delta-delta-Ct” (threshold cycle). Data were normalized relative to *HPRT* mRNA levels. The p-value was calculated using student test. **B**, Relative luciferase activity in HaCaT cells transfected either with control pGL3-basic vector or with pGL3-Mrkl-prom plasmid, and irradiated with 50 mJ/cm^2^ UVB or not. Each histogram bar represents the mean value obtained from two independent experiments and six replicates for each condition. Raw data of luciferase assays were normalized relative to the samples protein amounts. The p-value was calculated using student test.

We investigated changes in MCPyV mRNA levels following solar radiations administered to the P18 MCPyV-positive volunteer. Using quantitative RT-PCR with specific primer-sets (STA, LTA2 and VP1b) based on the previously identified genome-sequence, we amplified small T (ST), LT antigen and VP1 mRNA. Given that MCPyV mRNAs are less abundant than the human mRNAs, a pre-amplification step was included, as previously reported [19]. We showed that ST antigen mRNA levels were significantly higher in skin samples exposed to radiation at 4 J/cm^2^ than in non-irradiated samples or samples exposed to 2 J/cm^2^ radiation (Figure 4A). This effect was not observed for either LT antigen or VP1 mRNA. The *interleukin 6* (*IL6*) mRNA, an UV-inducible gene used as a positive control, was also significantly increased in a dose-dependent manner (Figure 4A) ([22], [23]. Comparable data were obtained with 3 independent pre-amplification experiments performed on at least 2 samples per skin area condition (0–2–4 J/cm2, leading to a total of 6 reactions per irradiated condition). Taken together, these results confirmed the specific effect of UV-irradiation on ST mRNA with a mean increase of 7, defined by the IddCtI value. The levels of induction of ST antigen observed were more variable than for the cellular gene (*IL6*) as suggested by the error-bars data. This effect is inherent to the pre-amplification process, when starting with small amount of mRNA [24], and in our case to potential variable numbers of infected cells analyzed per sample. However, we analyzed *HPRT* and *IL6* relative expression levels, which proved to be similar between pre-amplified and non-amplified samples (data not shown), to confirm that the pre-amplification protocol was reliable and reproducible as it has been previously shown [25]. We sequenced the amplified product on both strands to verify that the PCR products obtained for each positive amplification product corresponded solely to ST antigen as suggested by the dissociation curve profile (data not shown). The sequences obtained were identical and unique corresponding to the ST antigen, confirming the specificity of the PCR product.

### The MCPyV non-coding control region is UV-inducible *in vitro*

Polyomavirus gene transcription is driven by a bidirectional promoter located in the non-coding control region (NCCR) of the viral genome (White et al. 2009). Detection of increased ST antigen transcript levels in patient P18 following *in vivo* skin irradiation suggests that the activity of MCPyV early promoter is potentially regulated in a UV-dependent manner. To investigate this possibility, we performed an *in vitro* UV-assay, as previously reported for the JVC (Rutberg et el. 2003). Using P18 DNA sample, we cloned the NCCR sequence that drives MCPyV early gene transcription upstream from the luciferase reporter gene. We examined the UV responsiveness of the MCPyV early promoter-luciferase reporter following transfection of HaCaT human keratinocyte cell line. UVB irradiation was performed forty hours post-transfection with a dose of 50 mJ/cm^2^ and cells were harvested five hours after UVB irradiation [26], [27]. The results show that MCPyV regulatory region possess an intrinsic transcriptional activity in HaCat cells (compared to the empty vector) and that this activity was significantly induced after UVB irradiation. A mean value of 1.8 fold increase is obtained with two independent experiments and six replicates per condition (Fig.4B).

## Discussion

MCC is a highly aggressive skin cancer associated with older age, immunosuppression and sun exposure. MCC affect preferentially individuals with light-colored skin and develop in the face, the most sun-exposed anatomical site. Recently, infection with MCPyV and clonal-integration of the virus into the human genome of MCC strongly suggest that MCPyV participates in the pathogenesis of the tumor. However, the precise mechanism and steps involved between time of infection by MCPyV and the development of MCC, as the impact of solar exposure remain unknown. We thus analyzed non-pathogenic skins of healthy Caucasian volunteers and UV-irradiated area to address this question, making the hypothesis that MCPyV gene expression could possibly be regulated in a UV-dependant manner. The current *in vivo* study provides two major informations. MCPyV is present in its episomal form in infected but non-pathogenic skin and more importantly ST antigen mRNA is specifically increased by usual solar radiations. Due to the rarity of MCPyV in normal skin, we used an *in vitro* assay to confirm that the MCPyV early promoter is indeed UV inducible, five hours post-UV radiation. The level of induction was found to be dependent on both the dose of UV delivered and the time post-induction (data not shown), which suggest the presence of a regulated process.

Taken together our finding report that the ST antigen mRNA level is regulated in response to UV and that the NCCR is UV inducible, which suggest that the increase in ST antigen mRNA following UV irradiation is dependent on the transcriptional machinery. Because a common promoter drives the transcription of the ST and LT antigens, the absence of an increase of the LT antigen mRNA suggests a concomitant activation of the splicing machinery, leading to a specific increase in ST antigen transcript levels five hours after irradiation. SV40 studies have shown, LT antigen to be the major transforming protein involved in neoplasic processes, deregulating p53 and retinoblastoma family members, whereas ST antigen cooperates with SV40 LT antigen to induce cell transformation [28], [29]. SV40 ST antigen has also been shown to inhibit the protein phosphatases 2A (PP2A), promoting entry into S phase [30]. Given that MCPyV LT antigen is mutated after integration into the MCC tumor genome, it is possible that MCPyV ST antigen plays an unexpected major role in viral replication [31] and in MCC development upon stimulation by solar radiation.

UV rays can modulate and mediate events in viral biology [32]. In the PyV family, the JCV promoter is finely regulated in a UV-dependent manner [33], and UV-rays specifically alter LT and ST transcription to promote replication of murine polyoma virus [34], [35], [36]. UV rays have also been shown, through the activation of the stress-responsive p38 kinase, to modulate the activity of cellular transcription factors [37], [38]. The viral machinery to execute the viral transcriptional program and promote development of the virus may recruit these transcription factors. In this study, we demonstrate for the first time the biological impact of single solar radiation in MCPyV regulation, thus providing a potential explanation for the role of repetitive solar-exposure in the pathogenesis of MCPyV-positive MCC. Further studies will now be needed to elucidate the precise molecular mechanisms involved including *cis* and *trans* acting regulatory elements and to characterize the cross talks between cellular and viral machineries.

## Materials and Methods

### Ethics Statement

The Ethics Committee of Rennes Hospital, France, approved the study (CCPPRB N°04/36-517).

### Volunteers

We recruited twenty healthy female volunteers, with skin phototype II or III, as defined by the Fitzpatrick classification system [39]. Participants with an average age of 42 years (range, 38–60 years) had all been referred for abdominal plastic surgery to the plastic-surgery department of South Hospital, Rennes, France. No patient had received UV radiation during the previous two months, or had taken photosensitive compounds. Patients on medical treatment or with striae on the region of the skin to be excised were excluded from the study. Each volunteer was fully informed about the procedures to be carried out and gave written consent before taking part in the photo-biological study. The study took place in the dermatology department of the Pontchaillou University Hospital, Rennes.

### Photo-biological protocol - Skin irradiation

We used a UV polychromatic light source (Dermolum UM-W1, Müller Elektronik®, Moosinning, Germany), equipped with a Schott WG 305 filter, to generate solar-simulated radiation (SSR) containing 5% UVB and 95% UVA. The simulator radiance was 100 mW/cm^2^ (Müller Elektronik® dosimeter).

The twenty patients were divided into two subgroups: group 1 consisted of patients P1 to P4 and group 2 consisted of patients P5 to P20. Patients in group 1 were not exposed to SSR. Group 2 patients received SSR at doses of 2 J/cm^2^ (30 sec irradiation) or 4 J/cm^2^ (1 min irradiation) on distinct areas (10 cm^2^ each) of the abdomen five hours before plastic surgery. The dose of 2 J/cm^2^ was chosen because it corresponds to the minimal erythema dose (MED) of SSR for phototype II Caucasian skin. The irradiated zones were distant one from each other, and emitted SSR could not affect the surrounding skin that was hidden. Skin patches (∼10 mm^2^) of UV-exposed areas were taken immediately after abdominal surgery and transferred directly into RNA*later* (Qiagen) for further nucleic acid extraction. Non-irradiated skin samples were taken as controls. Around five skin samples were taken from each area (irradiated and non-irradiated) [40], [41].

### Total RNA extraction

Skin homogenization was carried out with the Precellys®−24 device (Bertin), using ceramic beads (1.4 mm Ø, CK14), in the presence of 350 µl lysis buffer (RA1 Macherey-Nagel) supplemented with 3.5 µl β-mercapto-ethanol. The device was set at a speed of 6300 rpm, with a cycle duration of 23 seconds and an interval time of 2 minutes between 2 cycles, at 4°C. Six cycles were required for complete homogenization. Tri-reagent (400 µl; Sigma) was added, followed by 150 µl chloroform. The aqueous phase was recovered, mixed with 500 µl of 70% ethanol and transferred to a NucleoSpin® RNA II column. RNA was recovered from the column following the manufacturer's instructions, although the wash volumes were increased (RA2 = 600 µl; RA3 = 500 µl). The protocol included a stringent DNAse-treatment of the RNA extracts. Recovered RNA was quantified using the Nanodrop 1000 spectrophotometer (Nanodrop Technology®, Cambridge, UK) and RNA integrity was assessed using a 2100 Bioanalyser (Agilent, Palo Alto, CA, USA).

### Tissue DNA extraction

Skin sample DNA extractions were performed using a Macherey-Nagel NucleoSpin® Tissue kit. Skin samples were prepared by chopping with a sterile scalpel followed by Proteinase K digestion overnight at 56°C. Purified DNA samples were quantified using the Nanodrop 1000 spectrophotometer (Nanodrop Technology®, Cambridge, UK).

### Polymerase-Chain Reaction (PCR)

PCR experiments were carried out using AmpliTaq Gold DNA Polymerase (Applied Biosystems). PCR products were obtained by amplification of DNA (25 ng) using 500 nM of each primer, under the following cycling conditions: 10 min at 94°C and 40 cycles of 30 sec at 94°C, 30°C at 60°C and 1 min at 72°C), with a final step of 10 min at 72°C.

### Reverse Transcription of extracted RNA

Reverse transcription was performed with 500 ng RNA using Applied Biosystems High Capacity Reverse Transcription kit in a final volume of 20 µl.

### Pre-Amplification

An initial step of amplification was performed with the cDNA samples obtained from reverse transcription using the Applied Biosystems PreAmp Taqman Mix. cDNA (100 ng) was pre-amplified through 14 cycles as described on manufacturer protocol, in the presence of a pool of primers (0.06 µM), leading to over 15,000 fold-amplification on average.

### Quantitative PCR

We carried out relative quantitative PCR using the 7900HT Fast Real-Time PCR System (Applied Biosystems) after a 1:5 dilution of the pre-amplified samples in 1X TE. MCPyV mRNA sequences were detected with primers STA, LTA2 and VP1b (specific to the *small t antigen* (STA), *large T antigen* (LTA2) and *VP1* (VP1b) transcripts). Additional primers were used to target *HPRT* and *Interleukin 6* transcripts. Amplifications were obtained using 600 nM of each primer and the SYBR Green PCR master mix (Applied Biosystems). Standard cycling conditions of 10 min at 95°C and 40 cycles (15 sec at 95°C, 1 min at 60°C) were used. Particular attention was given to the quality of the dissociation curves. Data were analyzed using the delta-delta Ct method [42]. Primer sequences are available under request.

### Cloning of Merkel cell polyomavirus (MCV) promoter sequence

MCPyV promoter sequence was amplified from DNA extract of patient P18 skin sample. Two PCR experiments (nested PCR) were carried out, both using Pfu DNA Polymerase (Promega). The first PCR was performed on patient P18 DNA (50 ng), using the following primers: Mrkl-seq9-F: 5′-ACTCCTGTGGTGGCACTTAGTT-3′ and Mrkl-seq-9R: 5′-TCAGAGGGATGTTGCCATAAC-3′. The resulting PCR product was purified with Nucleospin Extract II kit (Macherey-Nagel), according to the manufacturer's instructions. The second PCR was performed on the purified sample (10 ng), using the following primers including KpnI and HindIII restriction sites: MCV-promF: 5′-(TCTGGTACC)CCCCCATCCTGAAAAATAAA-3′ and MCR-promR: 5′-(TCTAAGCTT)TCTATATGCAGAAGGAGTTTGCAG-3′. The cycling conditions were: 2 min at 95°C and 37 cycles of 30 sec at 95°C, 30 sec at 60°C and 2 min/kb at 72°C, with a final step of 5 min at 72°C. The MCV promoter sequence was then cloned in pGL3-basic Luciferase Repoter Vector (Promega) between KpnI and HindII restriction sites. Relevant clones were sequenced previous use.

### Transfection, irradiation of HaCaT human keratinocyte cells and luciferase assays

HaCaT human keratinocyte cells were grown in DMEM medium (Gibco BRL, Invitrogen) supplemented with 5% Fetal Bovine Serum (PAA cell culture company) and 1% Penicillin-Streptomycin antibiotics (Gibco BRL, Invitrogen), at 37°C in a controlled atmosphere provided with 5% CO_2_. Twenty-four hours before transfection, 2.5×10^4^ cells were seeded in 12-well plates. Cell transfection (with pGL3-basic vector or PGL3-MCV-prom plasmid) was performed using Lipofectamine 2000 (Invitrogen), according to the manufacturer's protocol. Fourty hours later, cells were irradiated with 50 mJ/cm^2^ UVB using the Stratalinker apparatus [43]. Five hours after irradiation, cells were harvested using Dual Luciferase assay Lysis Buffer (Promega) and the resulting lysate was homogenized. Luciferase assays were performed on cell lysates using Dual Luciferase assay kit (Promega), following the manufacturer's instructions, and using FLUOstar Omega luminometer (BMG Labtech). For normalization of luciferase signals, the protein amount of each cell lysate was measured using Bradford assay (Bio-Rad). Each experiment is performed at least two times and each condition are performed six times.

### Statistics

Statistical tests were performed using Student T-tests (unilateral test with inequal variances).

## References

[1] Boulais N, Misery L (2007). Merkel cells.. J Am Acad Dermatol.

[2] Toker C (1972). Trabecular carcinoma of the skin.. Arch Dermatol.

[3] Lemos B, Nghiem P (2007). Merkel cell carcinoma: more deaths but still no pathway to blame.. J Invest Dermatol.

[4] Engels EA, Frisch M, Goedert JJ, Biggar RJ, Miller RW (2002). Merkel cell carcinoma and HIV infection.. Lancet.

[5] Miller RW, Rabkin CS (1999). Merkel cell carcinoma and melanoma: etiological similarities and differences.. Cancer Epidemiol Biomarkers Prev.

[6] Feng H, Shuda M, Chang Y, Moore PS (2008). Clonal integration of a polyomavirus in human Merkel cell carcinoma.. Science.

[7] Becker JC, Houben R, Ugurel S, Trefzer U, Pfohler C (2009). MC polyomavirus is frequently present in Merkel cell carcinoma of European patients.. J Invest Dermatol.

[8] Garneski KM, Warcola AH, Feng Q, Kiviat NB, Leonard JH (2009). Merkel cell polyomavirus is more frequently present in North American than Australian Merkel cell carcinoma tumors.. J Invest Dermatol.

[9] Kassem A, Schopflin A, Diaz C, Weyers W, Stickeler E (2008). Frequent detection of Merkel cell polyomavirus in human Merkel cell carcinomas and identification of a unique deletion in the VP1 gene.. Cancer Res.

[10] Varga E, Kiss M, Szabo K, Kemeny L (2009). Detection of Merkel cell polyomavirus DNA in Merkel cell carcinomas.. Br J Dermatol.

[11] Carter JJ, Paulson KG, Wipf GC, Miranda D, Madeleine MM (2009). Association of Merkel cell polyomavirus-specific antibodies with Merkel cell carcinoma.. J Natl Cancer Inst.

[12] Dalianis T, Ramqvist T, Andreasson K, Kean JM, Garcea RL (2009). KI, WU and Merkel cell polyomaviruses: a new era for human polyomavirus research.. Semin Cancer Biol.

[13] Touze A, Gaitan J, Maruani A, Le Bidre E, Doussinaud A (2009). Merkel cell polyomavirus strains in patients with merkel cell carcinoma.. Emerg Infect Dis.

[14] Wieland U, Mauch C, Kreuter A, Krieg T, Pfister H (2009). Merkel cell polyomavirus DNA in persons without merkel cell carcinoma.. Emerg Infect Dis.

[15] Katano H, Ito H, Suzuki Y, Nakamura T, Sato Y (2009). Detection of Merkel cell polyomavirus in Merkel cell carcinoma and Kaposi's sarcoma.. J Med Virol.

[16] Corre S, Mekideche K, Adamski H, Mosser J, Watier E (2006). In vivo and ex vivo UV-induced analysis of pigmentation gene expressions.. J Invest Dermatol.

[17] Seite S, Colige A, Deroanne C, Lambert C, Piquemal-Vivenot P (2004). Changes in matrix gene and protein expressions after single or repeated exposure to one minimal erythemal dose of solar-simulated radiation in human skin in vivo.. Photochem Photobiol.

[18] Tolstov YL, Pastrana DV, Feng H, Becker JC, Jenkins FJ (2009). Human Merkel cell polyomavirus infection II. MCV is a common human infection that can be detected by conformational capsid epitope immunoassays.. Int J Cancer.

[19] Sastre-Garau X, Peter M, Avril MF, Laude H, Couturier J (2009). Merkel cell carcinoma of the skin: pathological and molecular evidence for a causative role of MCV in oncogenesis.. J Pathol.

[20] Duncavage EJ, Zehnbauer BA, Pfeifer JD (2009). Prevalence of Merkel cell polyomavirus in Merkel cell carcinoma.. Mod Pathol.

[21] Andres C, Belloni B, Puchta U, Sander CA, Flaig MJ (2009). Prevalence of MCPyV in Merkel cell carcinoma and non-MCC tumors.. J Cutan Pathol.

[22] Averbeck M, Beilharz S, Bauer M, Gebhardt C, Hartmann A (2006). In situ profiling and quantification of cytokines released during ultraviolet B-induced inflammation by combining dermal microdialysis and protein microarrays.. Exp Dermatol.

[23] Kuhn M, Wolber R, Kolbe L, Schnorr O, Sies H (2006). Solar-simulated radiation induces secretion of IL-6 and production of isoprostanes in human skin in vivo.. Arch Dermatol Res.

[24] Mestdagh P, Feys T, Bernard N, Guenther S, Chen C (2008). High-throughput stem-loop RT-qPCR miRNA expression profiling using minute amounts of input RNA.. Nucleic Acids Res.

[25] Ciotti P, Garuti A, Ballestrero A, Cirmena G, Chiaramondia M (2009). Reliability and reproducibility of a RNA preamplification method for low-density array analysis from formalin-fixed paraffin-embedded breast cancer samples.. Diagn Mol Pathol.

[26] Garcin G, Le Gallic L, Stoebner PE, Guezennec A, Guesnet J (2009). Constitutive expression of MC1R in HaCaT keratinocytes inhibits basal and UVB-induced TNF-alpha production.. Photochem Photobiol.

[27] Latonen L, Jarvinen PM, Suomela S, Moore HM, Saarialho-Kere U, et al. Ultraviolet B radiation regulates cysteine-rich protein 1 in human keratinocytes.. Photodermatol Photoimmunol Photomed.

[28] Cheng J, DeCaprio JA, Fluck MM, Schaffhausen BS (2009). Cellular transformation by Simian Virus 40 and Murine Polyoma Virus T antigens.. Semin Cancer Biol.

[29] Moens U, Van Ghelue M, Johannessen M (2007). Oncogenic potentials of the human polyomavirus regulatory proteins.. Cell Mol Life Sci.

[30] Khalili K, Sariyer IK, Safak M (2008). Small tumor antigen of polyomaviruses: role in viral life cycle and cell transformation.. J Cell Physiol.

[31] Kwun HJ, Guastafierro A, Shuda M, Meinke G, Bohm A (2009). The minimum replication origin of merkel cell polyomavirus has a unique large T-antigen loading architecture and requires small T-antigen expression for optimal replication.. J Virol.

[32] Purdie KJ, Pennington J, Proby CM, Khalaf S, de Villiers EM (1999). The promoter of a novel human papillomavirus (HPV77) associated with skin cancer displays UV responsiveness, which is mediated through a consensus p53 binding sequence.. Embo J.

[33] Sadowska B, Barrucco R, Khalili K, Safak M (2003). Regulation of human polyomavirus JC virus gene transcription by AP-1 in glial cells.. J Virol.

[34] Ronai ZA, Weinstein IB (1988). Identification of a UV-induced trans-acting protein that stimulates polyomavirus DNA replication.. J Virol.

[35] Ronai ZA, Weinstein IB (1990). Identification of ultraviolet-inducible proteins that bind to a TGACAACA sequence in the polyoma virus regulatory region.. Cancer Res.

[36] Rutberg SE, Yang YM, Ronai Z (1992). Functional role of the ultraviolet light responsive element (URE; TGACAACA) in the transcription and replication of polyoma DNA.. Nucleic Acids Res.

[37] Corre S, Primot A, Baron Y, Le Seyec J, Goding C (2009). Target gene specificity of USF-1 is directed via p38-mediated phosphorylation-dependent acetylation.. J Biol Chem.

[38] Jinlian L, Yingbin Z, Chunbo W (2007). p38 MAPK in regulating cellular responses to ultraviolet radiation.. J Biomed Sci.

[39] Fitzpatrick TB (1988). The validity and practicality of sun-reactive skin types I through VI.. Arch Dermatol.

[40] Corre S, Galibert MD (2005). Upstream stimulating factors: highly versatile stress-responsive transcription factors.. Pigment Cell Res.

[41] Mouchet N, Adamski H, Bouvet R, Corre S, Courbebaisse Y (2010). In vivo identification of solar radiation-responsive gene network: role of the p38 stress-dependent kinase.. PLoS One.

[42] Fleige S, Walf V, Huch S, Prgomet C, Sehm J (2006). Comparison of relative mRNA quantification models and the impact of RNA integrity in quantitative real-time RT-PCR.. Biotechnol Lett.

[43] Corre S, Primot A, Sviderskaya E, Bennett DC, Vaulont S (2004). UV-induced expression of key component of the tanning process, the POMC and MC1R genes, is dependent on the p-38-activated upstream stimulating factor-1 (USF-1).. J Biol Chem.

